# Long‐Term Outcomes of Reduced‐Toxicity Conditioning Using 8‐Gray Total Body Irradiation, Fludarabine, and Cyclophosphamide in Children, Adolescents, and Young Adults With Hematological Malignancies

**DOI:** 10.1002/hon.70026

**Published:** 2024-12-14

**Authors:** Hirokazu Morokawa, Koichi Hirabayashi, Yu Furui, Eri Okura, Shoji Saito, Yozo Nakazawa

**Affiliations:** ^1^ Department of Pediatrics Shinshu University School of Medicine Matsumoto Japan

**Keywords:** 8‐Gy total body irradiation, child, hematological malignancy, reduced‐toxicity conditioning, safety, survival

## Abstract

Recent studies have indicated that total body irradiation (TBI)‐based reduced‐toxicity conditioning (RTC) may be a potential treatment modality, especially in adults with leukemia. However, its efficacy and safety in children with hematological malignancies remain unclear. To investigate the long‐term outcomes and safety of allogeneic hematopoietic stem cell transplantation (allo‐HSCT) using an 8‐Gray (Gy) TBI/fludarabine (FLU)/cyclophosphamide (CY) RTC in children with hematological malignancies. We included 66 consecutive patients with leukemia, lymphoma, or myelodysplastic syndrome in this retrospective cohort study. Participants were < 25 years old and received an 8‐Gy TBI/FLU/CY RTC regimen followed by the first allo‐HSCT at Shinshu University Hospital between March 2004 and March 2021. The 5‐year overall and relapse‐free survival probabilities were 88.2% and 76.5%, respectively, in the lymphoid malignancy group. The myeloid malignancy group had probabilities of 72.4% and 58.6%, respectively. The 5‐year cumulative incidences of relapse and non‐relapse mortality were 20.6% and 2.9%, respectively, in the lymphoid malignancy group. These incidences were 37.9% and 3.4%, respectively, in the myeloid malignancy group. All patients had engraftment without early relapse and none developed grade 5 regimen‐related toxicity within 28 days after allo‐HSCT. Nonetheless, two patients had congenital abnormalities caused by chromosomal aberrations and died without relapse. 8‐Gy TBI/FLU/CY RTC was safe in children with hematological malignancies, regardless of the donor source. However, safety concerns were noted in cases of chromosomal aberration‐induced congenital abnormalities. Additionally, patients in the lymphoid and myeloid malignancy groups had favorable prognoses.

## 1 Introduction

Myeloablative total body irradiation (TBI)‐based conditioning is widely used in children with acute lymphoblastic leukemia (ALL) undergoing allogeneic hematopoietic stem cell transplantation (allo‐HSCT). It is widely used because studies report that superior survival outcomes are achieved when performed in children having a very high risk of ALL [1, 2, 3, 4, 5]. However, survivors experience various long‐term sequelae following TBI [6, 7, 8, 9, 10, 11], hence, continuous efforts are being made to avoid this conditioning [12, 13]. Recently, Spyridonidis et al. compared the outcomes in adults with ALL, who underwent allo‐HSCT in their first complete remission (CR‐1). The outcomes were compared after a conventional 12‐Gray (Gy) TBI‐based conditioning or an 8‐Gy TBI‐based reduced‐toxicity conditioning (RTC). They concluded that 8‐Gy TBI‐based RTC was sufficient for adults with ALL [14]. On the other hand, chemotherapy‐based conditioning is currently preferred in children with acute myeloid leukemia (AML) because TBI‐based conditioning has not shown a survival advantage over chemotherapy‐based conditioning in retrospective studies [15, 16, 17]. However, recent studies reported the advantages of TBI‐based RTC for adults with AML [18, 19].

Recent studies have shown that this approach could possibly be a potential conditioning modality; however, its efficacy and safety remain unclear because only a few reports have used this conditioning in children. In 2004, we devised a TBI‐based RTC comprising 8‐Gy TBI, fludarabine (FLU), and cyclophosphamide (CY). We continued using this regimen in children, adolescents, and young adults (AYA) with hematological malignancies. Additionally, we conducted a study that included 31 children and AYA with hematological malignancies in 2014. They underwent allo‐HSCT after 8‐Gy TBI/FLU/CY RTC and results showed favorable outcomes [20]. However, the study's findings were limited because the primary diseases were highly heterogeneous; the number of patients in each disease group was small. Notably, 66 patients received allo‐HSCT after 8‐Gy TBI/FLU/CY RTC, with a median follow‐up period of 10.1 years. Therefore, in the present study, we aimed to report the long‐term outcomes of 8‐Gy TBI/FLU/CY RTC in children and AYA with hematological malignancies.

## 2 Materials and Methods

### 2.1 Study Design and Inclusion Criteria

We included 66 consecutive patients with leukemia, lymphoma, or myelodysplastic syndrome (MDS) in this pilot study. All patients were < 25 years old and received an 8‐Gy TBI/FLU/CY RTC regimen followed by their first allo‐HSCT at Shinshu University Hospital between March 2004 and March 2021. We obtained patient information up to March 31, 2024, for all patients who underwent allo‐HSCT, and data were collected retrospectively from patients' electronic medical records. This study was approved by the Research Ethics Committee of the Shinshu University School of Medicine (approval number: 4239) and conducted in accordance with the Declaration of Helsinki. The requirement for informed consent was waived because of the retrospective nature of the study, in which we analyzed existing data with no identifiable private information.

### 2.2 Treatment

The preparative conditioning regimen comprised TBI (2‐Gy/day, day −7 to −4), FLU (30 mg/m^2^/day, day −8 to −4), and CY (60 mg/kg/day, day −3 to −2). After allo‐HSCT, patients with central nervous system disease received additional craniospinal irradiation (CSI). Furthermore, each physician decided on the CSI doses based on the patient's condition. CSI was conducted between engraftment to day +180. For matched‐sibling donors, we started the graft‐versus‐host disease (GVHD) prophylactic regimen, which included continuous intravenous cyclosporine A on day −1, along with short‐term methotrexate administered on days +1, +3, and +6. However, continuous intravenous tacrolimus was administered from day −1 for the other donors. Immunosuppressive agents, including short‐term methotrexate, methylprednisolone, and mycophenolate mofetil, were concomitantly administered following the physician's preference. Furthermore, patients with ALL or lymphoma received granulocyte‐colony stimulating factor from day +5 to days when the neutrophil count exceeded 1500/μL. All patients received acyclovir (10 mg/kg/day) and micafungin (2 mg/kg/day) intravenously during the peritransplant period for prophylaxis against reactivation of both herpes simplex and varicella‐zoster virus and fungal infections. Notably, prophylaxis against bacterial infection (intravenous piperacillin for patients aged < 15 years old and oral levofloxacin for those aged ≥ 15 years), was initiated from day −1 for all patients. Intravenous gamma globulin was administered at 200–400 mg/kg every 2 weeks up to day +60 and then monthly for up to 1 year. Additionally, trimethoprim/sulfamethoxazole was administered after engraftment for prophylaxis against *Pneumocystis jirovecii* infection. All patients received continuous infusions of low‐dose heparin and oral ursodeoxycholic acid from day −5 to prevent sinusoidal obstruction syndrome.

### 2.3 Endpoints and Definitions

Probabilities of overall survival (OS) and relapse‐free survival (RFS) and cumulative incidences of relapse and non‐relapse mortality (NRM), were the primary endpoints for this study. The secondary endpoints were engraftment, incidences of regimen‐related toxicity (RRT) within day +28 and representative transplantation‐related complications within day +100, cumulative incidences of acute and chronic GVHD, and incidences of late effects. We defined OS as the time between allo‐HSCT and death or March 31, 2024. Additionally, RFS was defined as the time between allo‐HSCT and relapse, death, or March 31, 2024. NRM was defined as death during remission. Notably, death due to primary disease progression, irrespective of other causes was death after relapse. We assessed RRT during the period from conditioning to day +28 following allo‐HSCT according to the National Cancer Institute Common Terminology Criteria for Adverse Events (Version 5.0). All transplant events, excluding GVHD and hematological toxicities, were defined as RRT. Furthermore, neutrophil engraftment was defined as the first of three consecutive days of achieving an absolute neutrophil count of at least 500/μL. Platelet engraftment was defined as the first day a platelet count of 20 × 10^9^/L was achieved without platelet transfusion. Similarly, red blood cell engraftment was defined as the first day a hemoglobin level > 8 g/dL was achieved without red blood cell transfusion. The absence of blood cell count recovery on day +100 or hematopoietic reconstitution using autologous cells was referred to as engraftment failure. Furthermore, we evaluated hematopoietic chimerism using fluorescent in situ hybridization with probes specific to sex chromosomes and/or by analyzing DNA microsatellite polymorphisms using polymerase chain reaction to distinguish between donor and recipient bone marrow cells. Chimerism was assessed at 1, 2, and 3 months after allo‐HSCT and subsequently every 3–6 months for 2 years. Full donor chimerism was defined as a donor cell profile > 99%. Both acute and chronic GVHD were graded following previously published criteria [21, 22].

### 2.4 Statistical Analyses

The patient and disease characteristics in the entire cohort were summarized using descriptive statistics. Patient characteristics were compared using the Mann–Whitney *U* test for continuous variables and Fisher's exact test for categorical variables. We used the Kaplan–Meier method to estimate the probabilities of OS and RFS, and groups were compared using the log‐rank test. The cumulative incidences of relapse, NRM, acute and chronic GVHD, and engraftment were estimated using the cumulative incidence method to accommodate for competing risks, and the groups were compared using Gray's test. NRM was a competing event for relapse, whereas relapse was a competing event for NRM. Furthermore, death and relapse before day +100 were the competing events for acute GVHD and neutrophil, platelet, and red blood cell engraftment. However, for chronic GVHD, death, and relapse during the study period were the competing events. All statistical analyses were performed using EZR software [23], and statistical significance was set at *p* < 0.05 (two‐sided).

## 3 Results

### 3.1 Patients and Disease Characteristics

The characteristics of the 66 patients who underwent their first allo‐HSCT after an 8‐Gy TBI/FLU/CY RTC are presented in Table 1. The median age of the patients at the time of allo‐HSCT was 10.1 years, and there were 32 (48.5%) male patients in our cohort. Furthermore, 46 (70.0%) patients underwent allo‐HSCT during their CR‐1 or CR‐2, whereas 20 (30.0%) underwent it in non‐CR or with an untreated primary disease. The stem cell sources were bone marrow (*N* = 33), cord blood (*N* = 31), and peripheral blood (*N* = 2) of donors. Of these, 24 donors (36.4%) were related. Notably, 5 patients had central nervous system disease and received CSI between day +77 and day +178 after allo‐HSCT. Additional brain and spinal doses for CSI were 10‐ to 16‐Gy and 0‐ to 9.6‐Gy, respectively.

**TABLE 1:** Patient and transplant‐related characteristics.

Characteristic	Total (*N* = 66)	Lymphoid (*N* = 34)	Myeloid (*N* = 29)	*p*‐value
Median age at transplantation, years (range)	10.1 (0.8–22.9)	11.0 (1.0–22.9)	7.8 (0.8–18.7)	0.010
Sex				0.616
Male	32 (48.5)	18 (52.9)	13 (44.8)	
Female	34 (51.5)	16 (47.1)	16 (55.2)	
Median time from diagnosis to allo‐HSCT, months (range)	8.8 (2.0–93.8)	16.1 (4.7–93.8)	7.3 (2.0–30.4)	0.001
Diagnosis				
ALL	28 (42.4)			
AML	18 (27.3)			
MDS	9 (13.6)			
NHL	4 (6.1)			
MPAL	3 (4.6)			
JMML	2 (3.0)			
ALCL	2 (3.0)			
Disease status at allo‐HSCT				< 0.001
CR1	23 (34.8)	16 (47.1)	5 (17.2)	
CR2	23 (34.8)	17 (50.0)	5 (17.2)	
Untreated primary disease	8 (12.1)	0 (0)	8 (27.6)	
Primary induction failure	6 (9.1)	0 (0)	6 (20.7)	
Refractory relapse	6 (9.1)	1 (2.9)	5 (17.2)	
CNS disease				0.057
Yes	5	5	0	
No	61	29	29	
HCT‐CI score				0.395
0	59 (89.4)	31 (91.2)	25 (86.2)	
1	3 (4.5)	1 (2.9)	2 (6.9)	
2	1 (1.5)	1 (2.9)	0 (0)	
3	2 (3.0)	0 (0)	2 (6.9)	
4	0 (0)	0 (0)	0 (0)	
5	1 (1.5)	1 (2.9)	0 (0)	
More than 6	0 (0)	0 (0)	0 (0)	
Stem cell source				0.899
Bone marrow	33 (50.0)	18 (52.9)	14 (48.3)	
Cord blood	31 (47.0)	15 (44.1)	14 (48.3)	
Peripheral blood	2 (3.0)	1 (2.9)	1 (3.4)	
Related donor				0.294
Yes	24 (36.4)	10 (29.4)	13 (44.8)	
No	42 (63.6)	24 (70.6)	16 (55.2)	
GVHD prophylaxis				0.271
TAC + MTX + mPSL	9 (13.6)	3 (8.8)	5 (17.2)	
TAC + MMF + mPSL	1 (1.5)	0 (0)	1 (3.4)	
TAC + MTX	34 (51.5)	20 (58.8)	14 (48.3)	
TAC + mPSL	13 (19.7)	5 (14.7)	6 (20.7)	
TAC + MMF	4 (6.1)	4 (11.8)	0 (0)	
CyA + MTX	5 (7.6)	2 (5.9)	3 (10.3)	

*Note:* Data presented are *N* (%) unless otherwise indicated.

Abbreviations: ALCL, anaplastic large cell lymphoma; ALL, acute lymphoblastic leukemia; allo‐HSCT, allogeneic hematopoietic stem cell transplantation; AML, acute myeloid leukemia; CNS, central nervous system disease; CR, complete remission; CyA, cyclosporin A; GVHD, graft‐versus‐host disease; HCT‐CI, hematopoietic cell transplantation‐specific comorbidity index; JMML, Juvenile myelomonocytic leukemia; MDS, myelodysplastic syndrome; MMF, mycophenolate mofetil; MPAL, mixed phenotype acute leukemia; mPSL, methylprednisolone; MTX, methotrexate; NHL, non‐Hodgkin lymphoma; TAC, tacrolimus.

Additionally, the characteristics of the primary diseases, including lymphoid malignancies (ALL, non‐Hodgkin lymphoma, and anaplastic large cell lymphoma) and myeloid malignancies (AML, MDS, and juvenile myelomonocytic leukemia), were summarized in the present study. The lymphoid malignancy group had participants with older mean ages at the time of allo‐HSCT than the myeloid malignancy group (11.0 vs. 7.8 years, *p* = 0.010). The median time from diagnosis to allo‐HSCT was longer in the lymphoid malignancy group than in the myeloid malignancy group (16.1 vs. 7.3 months, *p* = 0.001). Moreover, the CR rate at the time of allo‐HSCT was lower in the myeloid malignancy group than in the lymphoid malignancy group (*p* < 0.001). Sex, hematopoietic cell transplantation‐specific comorbidity index score, donor, or GVHD prophylaxis did not differ between the two groups.

### 3.2 Survival, Relapse, Non‐Relapse Mortality, and Cause of Death

**FIGURE 1:**
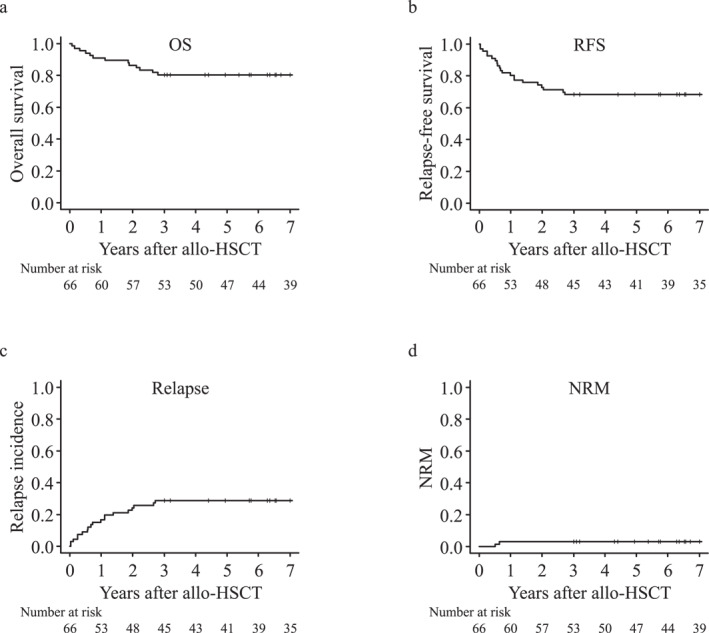
Outcomes in the entire cohort. (a) Probability of overall survival (OS). (b) Probability of relapse‐free survival (RFS). (c) Cumulative incidence of relapse. (d) Cumulative incidence of non‐relapse mortality (NRM).

**FIGURE 2:**
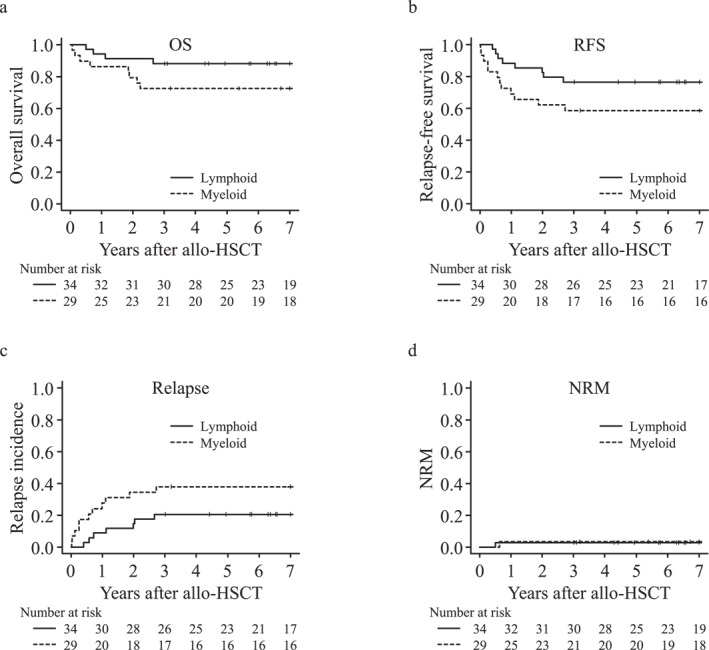
Outcomes according to primary disease. (a) Probability of overall survival (OS). (b) Probability of relapse‐free survival (RFS). (c) Cumulative incidence of relapse. (d) Cumulative incidence of non‐relapse mortality (NRM).

The probabilities of 5‐year OS and RFS were 80.3% and 68.2%, respectively, with a median follow‐up of 10.7 years for survivors in the entire cohort. Notably, the 5‐year cumulative incidence of NRM was low (3.0%). Approximately 14 patients had died due to relapse (*N* = 12), acute pneumonia (*N* = 1), and acute GVHD (*N* = 1) at the last follow‐up visit. The two patients who died without relapse had congenital abnormalities caused by chromosomal aberrations (trisomy 1q and 21). In addition, we compared the outcomes in the myeloid malignancy group by disease status before allo‐HSCT (Figure S1). The probability of the 5‐year OS in CR was significantly higher than that in non‐CR (100% vs. 57.9%, *p* = 0.022); however, the probability of 5‐year RFS did not differ between CR and non‐CR (70.0% vs. 52.6%, *p* = 0.227). Table 2, Figures 1 and 2, and Figure S1 presents the data regarding the survival, relapse, NRM, and cause of death.

**TABLE 2:** Transplant‐related events.

Characteristic	Total (*N* = 66)	Lymphoid (*N* = 34)	Myeloid (*N* = 29)	*p*‐value
Median follow‐up time after allo‐HSCT, year (range)	10.7 (3.0–20.1)	8.2 (3.0–19.7)	14.3 (3.2–20.1)	0.014
OS at 5 years, % (95% CI)	80.3 (68.5–88.1)	88.2 (71.6–95.4)	72.4 (52.3–85.1)	0.209
RFS at 5 years, % (95% CI)	68.2 (55.5–78.0)	76.5 (58.4–87.5)	58.6 (38.8–74.0)	0.094
Relapse at 5 years, % (95% CI)	28.8 (18.4–40.0)	20.6 (8.9–35.6)	37.9 (20.5–55.3)	0.096
NRM at 5 years, % (95% CI)	3.0 (0.6–9.5)	2.9 (0.2–13.2)	3.4 (0.2–15.3)	0.919
Median time of engraftment, days (range)				
Neutrophil	18 (9–42)	16 (9–32)	21.5 (16–42)	0.005
Platelet	25 (6–77)	24.5 (13–47)	29 (6–77)	0.245
Red blood cell	23 (9–74)	22 (9–54)	23.5 (10–74)	0.749
Graft failure	0 (0)	0 (0)	0 (0)	1.000
Acute GVHD at day +100, % (95% CI)				
Grades II–IV	48.5 (35.9–60.0)	50.0 (32.1–65.6)	44.8 (26.1–62.0)	0.926
Grades III–IV	22.7 (13.5–33.5)	17.6 (7.0–32.2)	24.1 (10.4–40.9)	0.458
Chronic GVHD at 5 years, % (95% CI)				
Limited + extensive	30.3 (19.6–41.6)	35.3 (19.7–51.3)	20.7 (8.2–37.1)	0.308
Extensive	12.1 (5.6–21.3)	14.7 (5.3–28.7)	10.3 (2.5–24.7)	0.606
Cause of death				
Primary disease	12 (18.2)	4 (11.8)	7 (24.1)	
Acute GVHD	1 (1.5)	1 (2.9)	0 (0)	
Acute pneumonia	1 (1.5)	0 (0)	1 (3.4)	

*Note:* Data presented are *N* (%) unless otherwise indicated.

Abbreviations: allo‐HSCT, allogeneic hematopoietic stem cell transplantation; CI, confidence interval; GVHD, graft‐versus‐host disease; NRM, non‐relapse mortality; OS, overall survival; RFS, relapse‐free survival.

### 3.3 Engraftment

**FIGURE 3:**
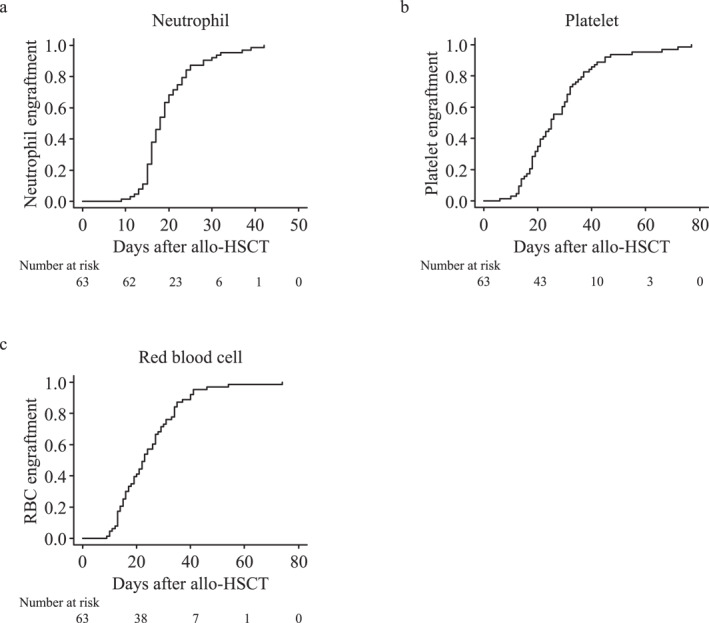
The cumulative incidences of neutrophil (a), platelet (b), and red blood cell (c) engraftment in the entire cohort.

We observed early hematological relapse within day +100 in three patients, and the remaining 63 achieved neutrophil, platelet, and red blood cell engraftment after a median of 18, 25, and 23 days, respectively. The engraftment results are shown in Table 2, Figure 3, and Figure S2. Furthermore, all 63 patients achieved full donor chimerism within day +100. Neutrophil engraftment occurred earlier in the lymphoid malignancy group than in the myeloid malignancy group (16 vs. 21.5 days, *p* = 0.005). The platelet and red blood cell engraftment time did not differ significantly between the two groups.

### 3.4 Toxicity and Graft‐Versus‐Host Disease

The RRTs within day +28 are presented in Table 3. Notably, the most common grade 3 RRT was febrile neutropenia (*N* = 58 [87.9%]). Furthermore, the grade 4 RRTs included elevated transaminase levels (*N* = 4 [6.1%]) and hyperbilirubinemia (*N* = 1 [1.5%]). None of the patients developed grade 5 RRT. Representative transplantation‐related complications within day +100 were observed as follows: sinusoidal obstruction syndrome in one patient, human herpesvirus six encephalitis in two patients, hemorrhagic cystitis in four patients, and acute pancreatitis in two patients, respectively.

**TABLE 3:** Regimen‐related toxicity within 28 days after allo‐HSCT.

Organ/system	Grade				
	1	2	3	4	5
Skin	9 (13.6)	0	0	0	0
Gastrointestinal					
Mucositis	23 (34.8)	6 (9.1)	5 (7.6)	0	0
Nausea/vomiting	15 (22.7)	4 (6.1)	23 (34.8)	0	0
Diarrhea	25 (37.9)	7 (10.6)	3 (4.5)	0	0
Hemorrhage					
Gastrointestinal	1 (1.5)	0	0	0	0
Urinary	3 (4.5)	3 (4.5)	1 (1.5)	0	0
Pulmonary	0	0	0	0	0
Infection					
Febrile neutropenia	0	0	58 (87.9)	0	0
Liver					
Transaminase elevation	15 (22.7)	17 (25.8)	17 (25.8)	4 (6.1)	0
Hyperbilirubiniemia	22 (33.3)	8 (12.1)	0	1 (1.5)	0
Kidney					
Creatinine elevation	2 (3.0)	1 (1.5)	0	0	0
Neurology					
Seizure	0	0	1 (1.5)	0	0

*Note:* Data presented are *N* (%).

Abbreviation: allo‐HSCT, allogeneic hematopoietic stem cell transplantation.

The cumulative incidences of grades III–IV acute GVHD and extensive chronic GVHD were 22.7% and 12.1%, respectively. The incidence of GVHD did not differ significantly between the lymphoid and myeloid malignancy groups. The toxicity and graft‐versus‐host disease data are presented in Tables 2 and 3.

### 3.5 Late Effects

During the study period, 18 patients developed late effects, with two patients experiencing two types of late effects. The following late effects were observed during the study period: hypogonadism in 11, short stature in three, thyroid cancer and hypothyroidism in two, and rhabdomyosarcoma and leiomyosarcoma in one patient(s).

## 4 Discussion

In the present study, we retrospectively evaluated the long‐term outcomes and safety of allo‐HSCT using 8‐Gy TBI/FLU/CY RTC in children and AYA with hematological malignancies. The findings from our study showed that 8‐Gy TBI/FLU/CY RTC was safe and provided excellent disease control in these populations. Furthermore, engraftment was robust and sustained, and full donor chimerism persisted even in cord blood recipients.

To date, 12‐Gy TBI‐based conditioning has been performed mainly for children with ALL. In the present study, the 5‐year OS, RFS, cumulative incidence of relapse, and cumulative incidence of NRM in the lymphoid malignancy group (including 28 patients with ALL) were 88.2%, 76.5%, 20.6%, and 2.9%, respectively. Comparing these outcomes to those of previous reports, we observed that they were better than those of Willasch et al. [5] and Davies et al. [2] however, almost similar to those of Peters et al. [3] Hence, a prospective comparative study is required to clarify whether TBI dose can be reduced from 12‐ to 8‐Gy in children with ALL.

Additionally, chemotherapy‐based conditioning is widely used in children with AML, and recent studies reported favorable outcomes in younger adults with AML after 8‐Gy TBI‐based conditioning [18, 19]. In the present study, the 5‐year OS, RFS, cumulative incidence of relapse, and NRM in 29 patients in the myeloid malignancy group (including 18 patients with AML) were 72.4%, 58.6%, 37.9%, and 3.4%, respectively. Considering that only 19 children (65.5%) were in non‐CR, our results seem to be as favorable as those in previous reports. However, more cases of patients receiving 8‐Gy TBI‐based RTC are required to clarify whether 8‐Gy TBI‐based RTC is a potential conditioning modality for children with AML.

Moreover, there were only two NRM cases, during the study period. Furthermore, the two patients who died without relapse had congenital abnormalities caused by chromosomal aberrations. These results suggested that the safety of 8‐Gy TBI/FLU/CY RTC was high. However, because patients with congenital abnormalities caused by chromosomal aberrations tended to develop severe RRTs even with 8‐Gy TBI/FLU/CY RTC in the present study, lower‐intensity conditioning, and more careful follow‐up may be required for children with congenital abnormalities caused by chromosomal aberrations.

In addition, graft failure is a crucial complication associated with an increased incidence of NRM. Reduction in the intensity of conditioning and receiving cord blood are risk factors for this failure [24]. In the present study, 31 patients (47.0%) received cord blood; however, no graft failure was observed in any of the 63 patients, except for three who had an early relapse within day +100. This result suggests that 8‐Gy TBI/FLU/CY RTC is sufficient to achieve engraftment in children with hematological malignancies, regardless of the donor.

For patients who are cured of their original disease and survive long‐term after allo‐HSCT, late effects are major concerns [6, 7, 8, 9, 10, 11]. In the present study, 18 patients developed late effects during the study period, with an incidence of 40.0% observed among patients who survived long‐term. Considering that late effects will increase with the lapse of time, this incidence is not satisfactory. Notably, four patients developed secondary malignancies. Considering that secondary malignancies have been reported to be more frequent after TBI than after irradiation‐free conditioning regimens regardless of the TBI dose [25, 26], careful follow‐up of secondary malignancies is important for patients who received 8‐Gy TBI/FLU/CY RTC. In addition, a direct comparison between 12‐ and 8‐Gy TBI is required to clarify whether reduction of TBI dose can reduce the risk of secondary malignancies.

This study has some limitations. First, we included patients with a wide variety of diseases and disease statuses in our cohort. In the myeloid malignancy group, 65.5% of the patients were in non‐CR before allo‐HSCT. Therefore, it is difficult to compare the outcomes of the present study with those of previous studies. Second, it was difficult to accurately assess the efficacy and safety of 8‐Gy TBI‐based conditioning because our study was retrospective and non‐comparative. Therefore, prospective comparable studies are required to obtain more reliable evidence.

## 5 Conclusion

In conclusion, 8‐Gy TBI/FLU/CY RTC was safe in children and AYA with hematological malignancies, regardless of the donor source. In addition, the prognoses, including OS and RFS, were favorable in patients in the lymphoid malignancy and myeloid malignancy groups.

## Author Contributions

**Hirokazu Morokawa:** data curation, formal analysis, investigation, methodology, resources, visualization, writing–original draft, writing–review and editing. **Koichi Hirabayashi:** conceptualization, formal analysis, investigation, methodology, project administration, visualization, writing–original draft, writing–review and editing. **Yu Furui:** resources, writing–review and editing. **Eri Okura:** resources, writing–review and editing. **Shoji Saito:** resources, writing–review and editing. **Yozo Nakazawa:** resources, writing–review and editing. All authors interpreted the results, reviewed the manuscript, participated actively in drafting the final version of the paper, and approved the final manuscript.

## Ethics Statement

This study was approved by the Research Ethics Committee of the Shinshu University School of Medicine (approval number: 4239) and conducted in accordance with the Declaration of Helsinki.

## Consent

The requirement for informed consent was waived because of the retrospective nature of the study, in which we analyzed existing data with no identifiable private information.

## Conflicts of Interest

The authors declare no conflicts of interest.

### Peer Review

The peer review history for this article is available at https://www.webofscience.com/api/gateway/wos/peer-review/10.1002/hon.70026.

## Supporting information

Figures S1–S2

## Data Availability

The data that support the findings of this study are available from the corresponding author upon reasonable request.
