# Supplementary material for: Long‐Term Outcomes of Reduced‐Toxicity Conditioning Using 8‐Gray Total Body Irradiation, Fludarabine, and Cyclophosphamide in Children, Adolescents, and Young Adults With Hematological Malignancies
Source: Hematol Oncol. 2024 Dec 14;43(1):e70026. doi: 10.1002/hon.70026 (PMC11646108; doi:10.1002/hon.70026)
Supplement: Supplementary file 1 — Figures S1–S2 [file HON-43-e70026-s001.pptx]

## Slide 1
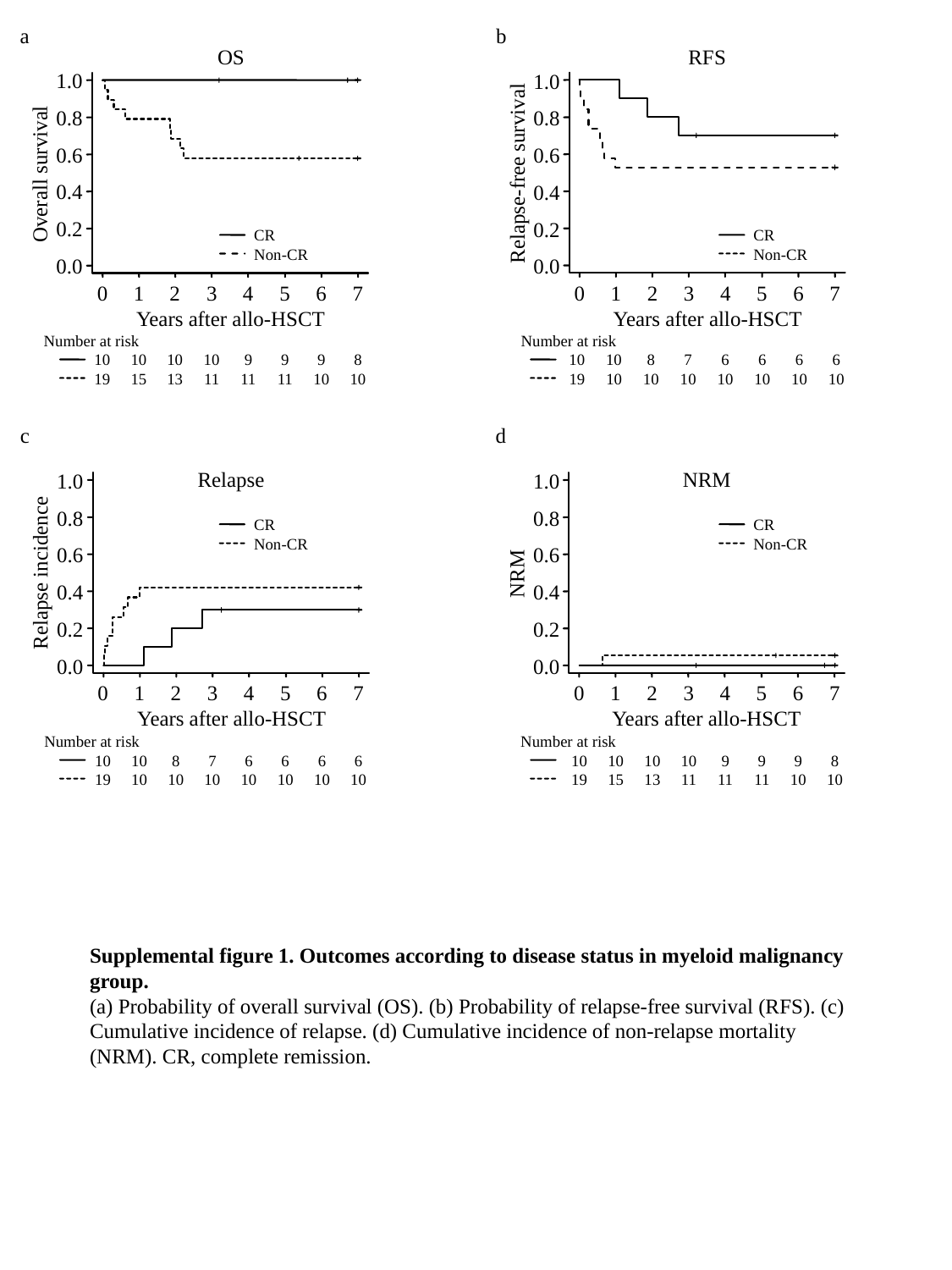

a
b
OS
RFS
1.0
1.0
0.8
0.8
0.6
0.6
Relapse-free survival
Overall survival
0.4
0.4
0.2
0.2
CR
CR
Non-CR
Non-CR
0.0
0.0
0
1
2
3
4
5
6
7
0
1
2
3
4
5
6
7
Years after allo-HSCT
Years after allo-HSCT
Number at risk
Number at risk
10
10
10
10
9
9
9
8
10
10
8
7
6
6
6
6
19
15
13
11
11
11
10
10
19
10
10
10
10
10
10
10
c
d
1.0
1.0
Relapse
NRM
0.8
0.8
CR
CR
Non-CR
Non-CR
0.6
0.6
Relapse incidence
NRM
0.4
0.4
0.2
0.2
0.0
0.0
0
1
2
3
4
5
6
7
0
1
2
3
4
5
6
7
Years after allo-HSCT
Years after allo-HSCT
Number at risk
Number at risk
10
10
8
7
6
6
6
6
10
10
10
10
9
9
9
8
19
10
10
10
10
10
10
10
19
15
13
11
11
11
10
10
Supplemental figure 1. Outcomes according to disease status in myeloid malignancy group.
(a) Probability of overall survival (OS). (b) Probability of relapse-free survival (RFS). (c) Cumulative incidence of relapse. (d) Cumulative incidence of non-relapse mortality (NRM). CR, complete remission.

## Slide 2
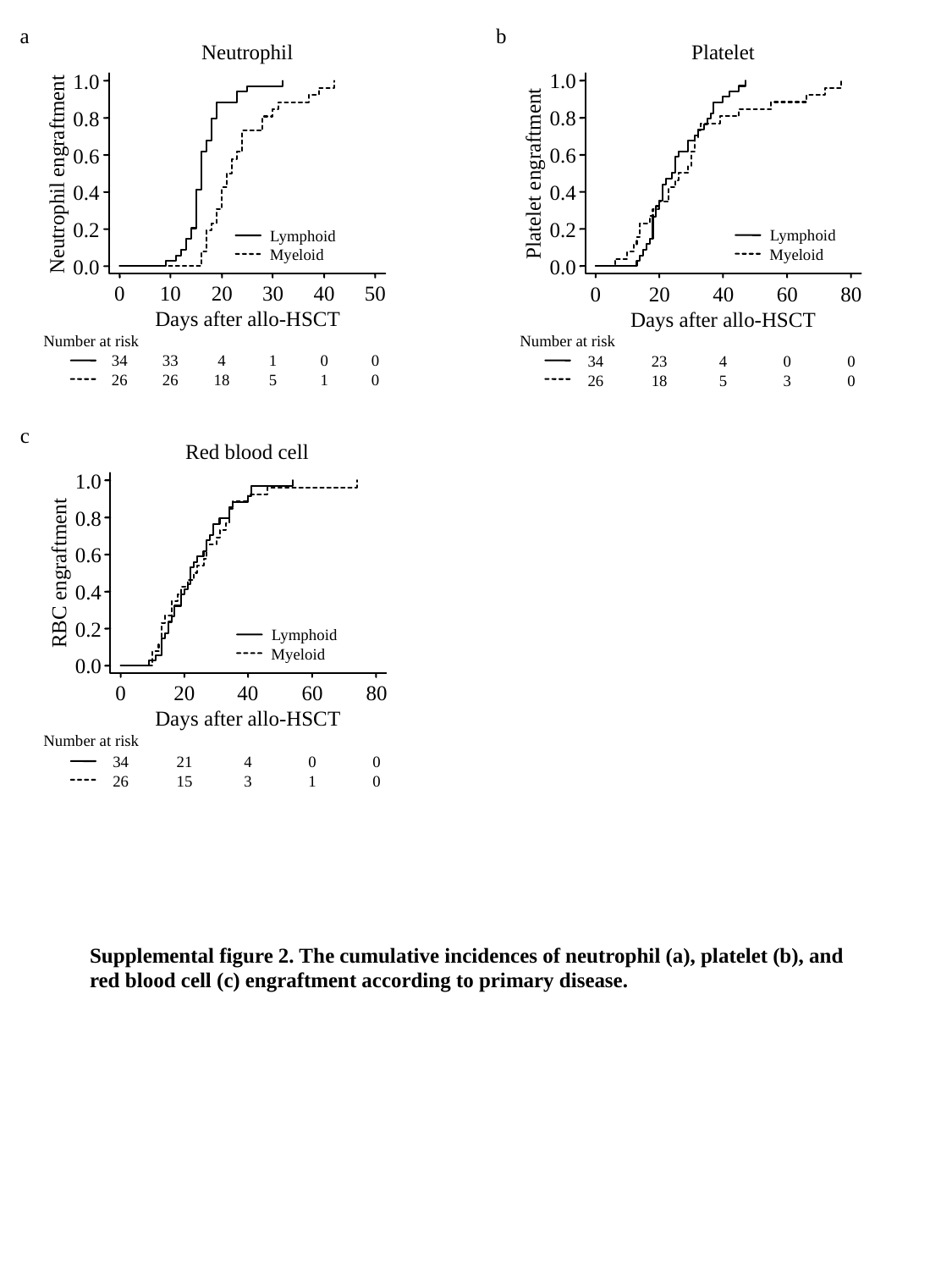

a
b
Neutrophil
Platelet
1.0
1.0
0.8
0.8
0.6
0.6
Platelet engraftment
Neutrophil engraftment
0.4
0.4
0.2
0.2
Lymphoid
Lymphoid
Myeloid
Myeloid
0.0
0.0
0
10
20
30
40
50
0
20
40
60
80
Days after allo-HSCT
Days after allo-HSCT
Number at risk
Number at risk
34
33
4
1
0
0
34
23
4
0
0
26
26
18
5
1
0
26
18
5
3
0
c
Red blood cell
1.0
0.8
0.6
RBC engraftment
0.4
0.2
Lymphoid
Myeloid
0.0
0
20
40
60
80
Days after allo-HSCT
Number at risk
34
21
4
0
0
26
15
3
1
0
Supplemental figure 2. The cumulative incidences of neutrophil (a), platelet (b), and red blood cell (c) engraftment according to primary disease.
